# Pediatric dental cone-beam computed tomography using half-acquisition and low-noise reconstruction: visual evaluation of clinical images

**DOI:** 10.1007/s13246-025-01691-2

**Published:** 2026-01-08

**Authors:** Misaki Ito, Ikuho Kojima, Masahiro Iikubo, Shu Onodera, Masahiro Sai, Masaki Fujisawa, Toshiki Kato, Masaaki Nakamura, Masayuki Zuguchi, Koichi Chida

**Affiliations:** 1 Department of Radiology, Tohoku University Hospital, 1-1 Seiryo-machi, Aoba-ku, Sendai, 980-8574 Japan; 2 Department of Radiological Technology, Tohoku University Graduate School of Medicine, 2-1 Seiryo-machi, Aoba-ku, Sendai, 980-8575 Japan; 3 Division of Oral and Maxillofacial Radiology, Tohoku University Hospital, 1-1 Seiryo-machi, Aoba-ku, Sendai, 980-8574 Japan; 4 Division of Dental Informatics and Radiology, Tohoku University Graduate School of Dentistry, 4-1 Seiryo-machi, Aoba-ku, Sendai, 980-8575 Japan; 5 Division of Disaster Medical Science, International Research Institute of Disaster Science, Tohoku University, 468-1 Aoba, Aramaki, Aoba-ku, Sendai, 980-8572 Japan

**Keywords:** Dental cone-beam computed tomography, Child, Rotation angle, Reconstruction filter, Radiation dose, Clinical image evaluation

## Abstract

This study evaluated whether half-acquisition (180° scan) pediatric cone-beam computed tomography (CBCT; 3D Accuitomo F17, J. Morita, Kyoto, Japan) reduces radiation exposure while maintaining sufficient diagnostic image quality for identifying ectopic eruptions and impacted teeth. Additionally, it was investigated whether a low-noise reconstruction filter (G_101) mitigates image quality degradation in 180° scans. Three board-certified oral and maxillofacial radiologists certified by the Japanese Society for Oral and Maxillofacial Radiology visually evaluated clinical images from 12 pediatric patients (aged 6–10 years). The image quality was objectively assessed using phantom-based analyses of the modulation transfer function (MTF), noise power spectrum (NPS), and comprehensive objective image quality calculated from MTF and NPS values. Although 180° images showed increased noise and slightly lower visual assessment scores compared with 360° images, they remained diagnostically acceptable. In 180° reconstructions, the median visual scores with the G_101 filter were slightly higher than those with the standard G_001 filter, with small differences (within approximately 0–3 points on a 100-point scale), although the differences were not statistically significant. Interestingly, in approximately 28% of 180 evaluations, 180° images scored higher than 360° images, likely due to reduced motion artefacts from shorter acquisition. In a previous phantom experiment, the dose area product (DAP) for 360° and 180° scans was 490 mGy cm^2^ and 249 mGy cm^2^, respectively, indicating that 180° scan reduces radiation exposure while maintaining clinically acceptable image quality. These findings suggest that half-acquisition, when combined with an appropriate reconstruction filter, may offer a practical, low-dose alternative for pediatric dental imaging.

**Supplementary Information:**

The online version contains supplementary material available at 10.1007/s13246-025-01691-2.

## Introduction

Cone-beam computed tomography (CBCT) is a radiographic technique that uses a cone beam to construct three-dimensional images. It has been widely used in dentistry, particularly for ectopic and impacted teeth, implant planning, and orthodontics [1–4].

The most common diagnostic indications for dental CBCT in pediatric patients are ectopic eruptions and impacted teeth [5–7]. These examinations are performed in children as young as 4 years, with the most common age being 9 years [8, 9].

The justification and optimization of radiological examinations in pediatric patients are crucial, because children are more susceptible to the effects of ionizing radiation than adults [10–15]. Many dental CBCT scanners use variable rotation angles to reduce radiation doses. Compared with 360° scan, 180° scan can reduce the effective dose by approximately half, assuming that all other exposure parameters, such as tube current, are kept constant, especially when radiosensitive organs such as the eyes and thyroid gland are in the primary beam [16–19].

Pediatric patients are more likely to move during CBCT scanning, which can result in motion artifacts that degrade image quality [20]. According to Spin-Neto et al. [21], among 56 pediatric patients aged 9–15 years, 9 cases (16%) required repeat imaging due to patient movement.

Reducing the rotation angle may be particularly effective in pediatric patients. However, since this approach can compromise image quality by decreasing the amount of projection data and increasing image noise, the utility of the resulting images for diagnosis must be assessed. To the best of our knowledge, no previous study has evaluated clinical images of pediatric patients while varying the rotation angle.

Many currently available devices can change reconstruction filters, providing an opportunity to improve image quality without increasing radiation exposure. This is particularly important in pediatric imaging, where minimizing dose while maintaining diagnostic quality is essential. In this study, we used the 3D Accuitomo F17 (J. Morita Corporation, Kyoto, Japan), which provides a low-noise reconstruction filter (G_101). However, despite this advantage, no studies have evaluated the quality of pediatric clinical images after changing the reconstruction filter. Our previous phantom study [22] demonstrated that the periodontal ligament space showed the lowest visibility among dental structures in CBCT images, primarily due to image noise, suggesting that noise reduction is particularly important in pediatric imaging. Therefore, using a low-noise reconstruction filter, such as G_101, may help compensate for image degradation associated with reduced-angle acquisition.

In this context, the present study evaluated the effects of half-acquisition pediatric dental CBCT on reducing radiation exposure while maintaining sufficient diagnostic image quality to detect ectopic eruptions and impacted teeth. We also examined whether image quality degradation associated with reduced-angle acquisition could be mitigated by applying a low-noise reconstruction filter (G_101), based on subjective visual assessments and objective phantom-based analyses.

## Methods

This retrospective study was approved by the Ethics Committee of Tohoku University Graduate School of Dentistry (Approval No. 39430). The requirement for written informed consent from patients was waived because study information was disclosed through an opt-out document. Written informed consent was obtained from all observers who participated in the image evaluation.

### CBCT scanner

**Fig. 1:**
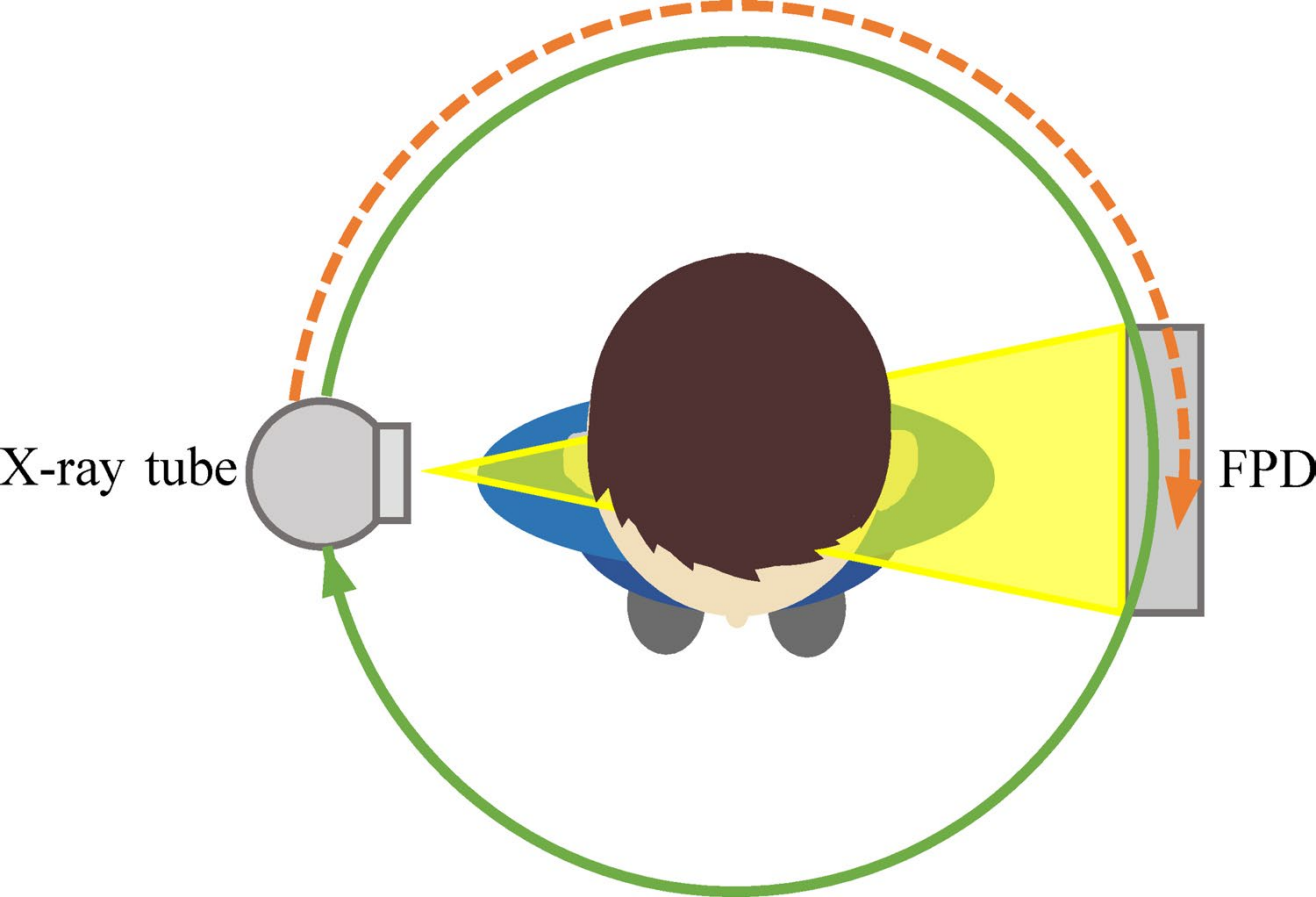
Schematic illustration of X-ray irradiation in CBCT. **a** 360° scan (green solid line): the X-ray tube moves at a 360° angle. **b** 180° scan (red dotted line): the X-ray tube moves at a 180° + fan angle. FPD flat-panel detector

This study was performed using a 3D Accuitomo F17 instrument (J. Morita Corporation) equipped with a cesium iodide (CsI) indirect-conversion flat-panel detector. The dental CBCT instrument offers gantry rotations of 360° or 180°. The exposure times were 17.5 s for 360° scan and 9.0 s for 180° scan (standard-dose mode). At 180° scan, the X-ray tube was rotated around the posterior aspect of the patient’s head. Only the standard exposure mode was available on this unit (high-speed mode not supported). Figure 1 shows a schematic illustration of X-ray irradiation in CBCT.

### Scanning and reconstruction

During the physical evaluation using phantom images, CT data were acquired at both 180° and 360° scan angles, with the center of rotation set at the center of the Sedentex CT-IQ phantom.

Clinical images were obtained using a 360° scan and retrospectively reconstructed to 180° images by extracting the projection data corresponding to the posterior 180° scan range (Fig. 1), as described by Apostolopoulos et al. [23]. A preliminary phantom experiment indicated that 180° images reconstructed from 360° scans were largely comparable to directly acquired 180° scans under identical conditions (details in Supplementary Information). We acknowledge that this reconstruction images may differ from the vendor’s native 180° scan, which is optimized for half-acquisition. The center of rotation was set at the anatomical region of the ectopic eruptions and the impacted teeth to ensure optimal image quality around the target region and to minimize unnecessary radiation exposure to surrounding tissues, as is commonly performed in clinical CBCT protocols.

The other scan and reconstruction parameters were consistent across both evaluations and are summarized in Table 1.

**Table 1:** Imaging conditions and image reconstruction conditions

Imaging conditions	Tube voltage (kV)	90
	Tube current (mA)	3
	Field of view (cm)	6 × 6
Image reconstruction conditions	Slice thickness (mm)	0.375
	Slice increment (mm)	0.125

Based on previous studies indicating that the periodontal ligament space exhibits low visibility owing to image noise [22], the reconstruction filters selected were the standard filter (G_001) and the low-noise filter (G_101).

### Phantom-based physical evaluation of reconstruction filters for 360°(G_001), 180°(G_001), and 180°(G_101)

#### Spatial resolution

Modulation Transfer Function (MTF) was obtained using the commonly adopted wire method. A Sedentex CT-IQ cylindrical phantom (Leeds Test Objects, Boroughbridge, UK), specifically designed for dental CBCT evaluation, was used. The phantom included a cylindrical point spread function insert (35 mm diameter, 20 mm height) containing a centrally positioned stainless-steel wire 0.25 mm in diameter. The rotation center was set to the steel wire, and dental CBCT scans were performed three times for each imaging condition.

The radial virtual slit method was used to analyze the MTF. MTF values were obtained from the slice image at the center of each acquisition, as well as from slices 0.375 mm anterior and posterior to the center. In total, MTF results from nine slices were summed and averaged. The acquired image data were exported in DICOM format and analyzed using CTmeasure version 0.98f [24].

The following three image sets were compared: 360°(G_001), 180°(G_001), and 180°(G_101). This comparison aimed to determine whether G_101 could compensate for the reduction in image quality typically observed in 180° scans, particularly in the context of detecting low-contrast structures such as the periodontal ligament space, which is especially vulnerable to low-frequency noise.

#### Noise texture

Noise Power Spectrum (NPS) was used to assess image noise texture. The lower section of the Sedentex CT-IQ phantom composed of uniform polymethyl methacrylate (density 1.20 ± 1.00%), was imaged. The center of rotation was set to the center of the phantom, and dental CBCT scans were performed three times for each imaging condition.

The radial frequency method was applied to analyze the NPS. A region of interest of 256 × 256 pixels was placed at the center of a 6 × 6 cm field of view (FOV) image. NPS results were obtained for 100 consecutive images (300 images in total) per scan, which were then summed and averaged. The acquired image data were exported in DICOM format and analyzed using CTmeasure version 0.98f [24].

Three image sets were compared: 360°(G_001), 180°(G_001), and 180°(G_101).

### Comprehensive objective image quality calculated from MTF and NPS values

The system performance (SP) function [25–28] was used as a comprehensive evaluation index for image quality. SP is calculated using the MTF and NPS values and is correlated with visual detection rates. The SP function is calculated using the following equation:

1 $$SP\left( f \right)^{2} = \frac{{MTF\left( f \right)^{2} }}{NPS\left( f \right)}$$

Where *f* is the spatial frequency.

### Visual evaluation using clinical images for 360°(G_001), 180°(G_001), and 180°(G_101)

#### Visual evaluation protocol

Visual evaluation was used to compare the quality of the 360° and 180° clinical images reconstructed from the original 360° datasets, as described in Sect. “Scanning and reconstruction”. A preliminary phantom study confirmed comparable spatial resolution and noise between 180° images reconstructed from 360° data and directly acquired 180° scans, thus validating their use in this visual evaluation.

The participants were consecutive pediatric patients aged 6–10 years who underwent dental CBCT examinations (90 kV, 3 mA, 6 × 6 cm FOV, and 360°, 17.5 s) to diagnose ectopic eruptions and impacted maxillary anterior teeth at Tohoku University Hospital between May 2020 and May 2023. Of the initial 21 participants, eight with incomplete roots and one with poor positioning were excluded. Thus, 12 patients were included in the visual evaluation (age range, 6–10 years; mean age, 7.33 years; eight boys and four girls). For each participant images, a 180° scan was generated from the original 360° scan using an i-Dixel diagnostic imaging workstation (J. Morita Corporation). Three types of images were created for visual evaluation per participant: 360° with the standard reconstruction filter (G_001), 180° with the standard filter (G_001), and 180° with the low-noise filter (G_101), enabling comparison across different rotation angles and reconstruction filters.

12 patients were visually evaluated by three oral and maxillofacial radiologists, board-certified by the Japanese Society for Oral and Maxillofacial Radiology, with an average of 24.6 years of experience (range 12–41 years). The radiologists scored each image on a 100-point scale. A score of 50 points was set as the minimum threshold for diagnostic acceptability. The evaluation included five key items necessary for diagnosing ectopic eruptions and impacted teeth [29]:

- Tooth position relative to the surrounding teeth.
- Tooth crown.
- Tooth root.
- Root apex of the tooth.
- Periodontal ligament space of the tooth.

Motion artifacts were not evaluated separately, but their impact is indirectly reflected in anatomical visibility. All images were reviewed on medical-grade liquid–crystal displays (RadiForce RX440, EIZO Co., Ltd., Ishikawa, Japan). Images were presented individually in random order. Observers were allowed to freely adjust window width and level settings, and no time limit was imposed for image evaluation.

### Statistical analysis

Intraclass correlation coefficients (ICCs) were calculated to assess both inter and intra-rater reliability. The inter-rater reliability was evaluated for all three imaging conditions (360°(G_001), 180°(G_001), and 180°(G_101)) using a single evaluation session for all 12 cases. Owing to time and workload constraints, intra-rater reliability was assessed only for 360°(G_001) and 180°(G_001), based on two randomly selected cases evaluated twice by each rater. Intra-rater reliability was not assessed for 180°(G_101). Significant differences in scores among the three image types were evaluated by pairwise comparisons using the Steel–Dwass test. All statistical analyses were performed using JMP Pro version 17.0.0 (SAS Institute Inc., Cary, NC, USA), with significance set at *p* < 0.05. ICC values of > 0.75, 0.40–0.75, and < 0.40 indicated excellent, fair to good, and poor reliability, respectively [30, 31].

## Results

### Phantom-based physical evaluation of reconstruction filters for 360°(G_001), 180°(G_001), and 180°(G_101)

#### Spatial resolution

**Fig. 2:**
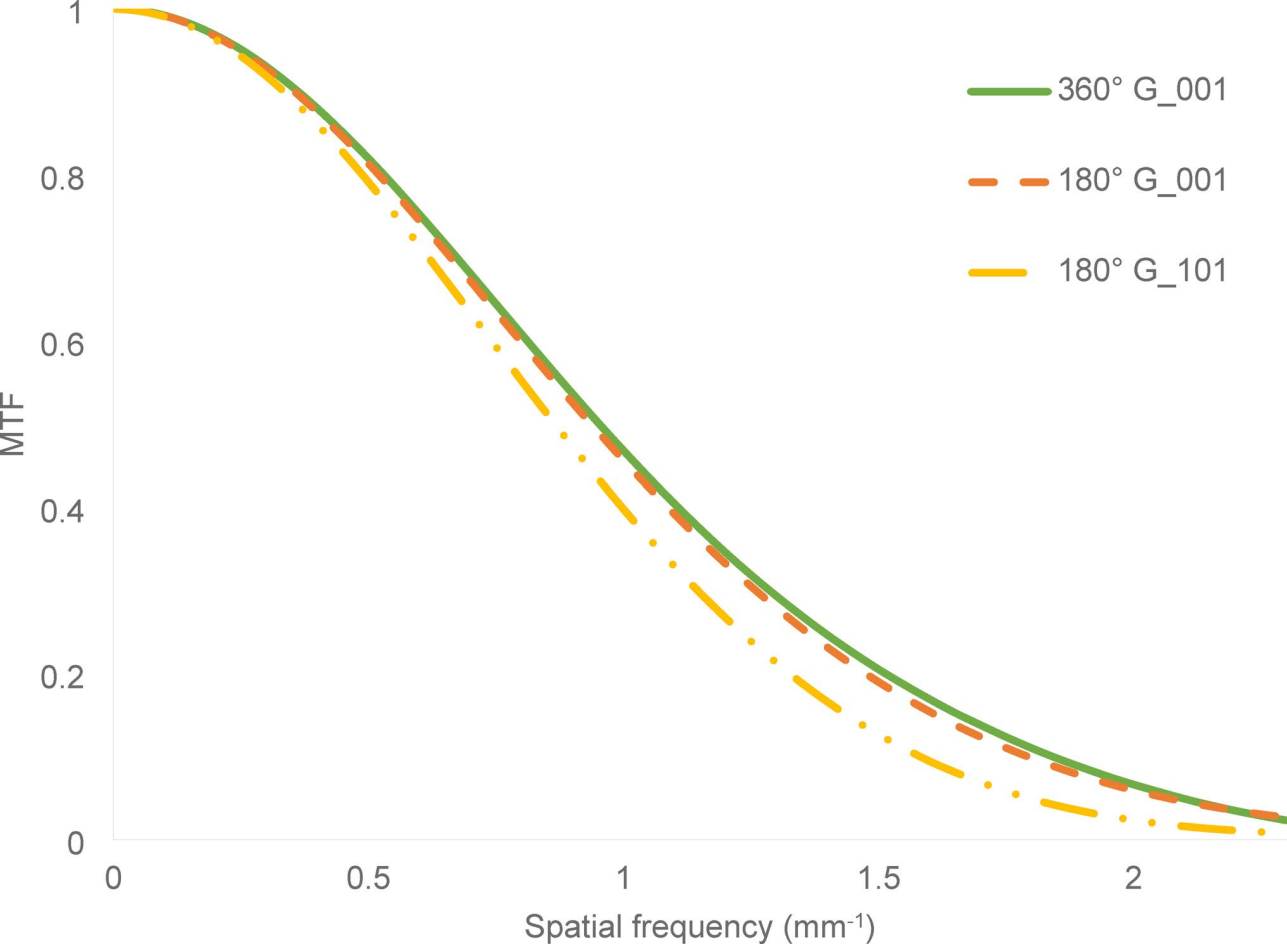
Comparison of MTF curves for 360°(G_001), 180°(G_001), and 180°(G_101). G_001 standard reconstruction filter, G_101 low-noise filter

The MTF curves for 360°(G_001), 180°(G_001), and 180°(G_101) were derived (Fig. 2). The 10% MTF values for each condition are shown in Table 2. The MTF curves were similar between 360°(G_001) and 180°(G_001). However, 180°(G_101) exhibited a lower 10% MTF frequency.

**Table 2:** 10% MTF and peak NPS values for 360°(G_001), 180°(G_001), and 180°(G_101)

Imaging condition	10% MTF (mm^−1^)	Peak NPS (mm^−1^)
360° G_001	1.799	0.643
180° G_001	1.758	0.694
180° G_101	1.583	0.537

#### Noise texture

**Fig. 3:**
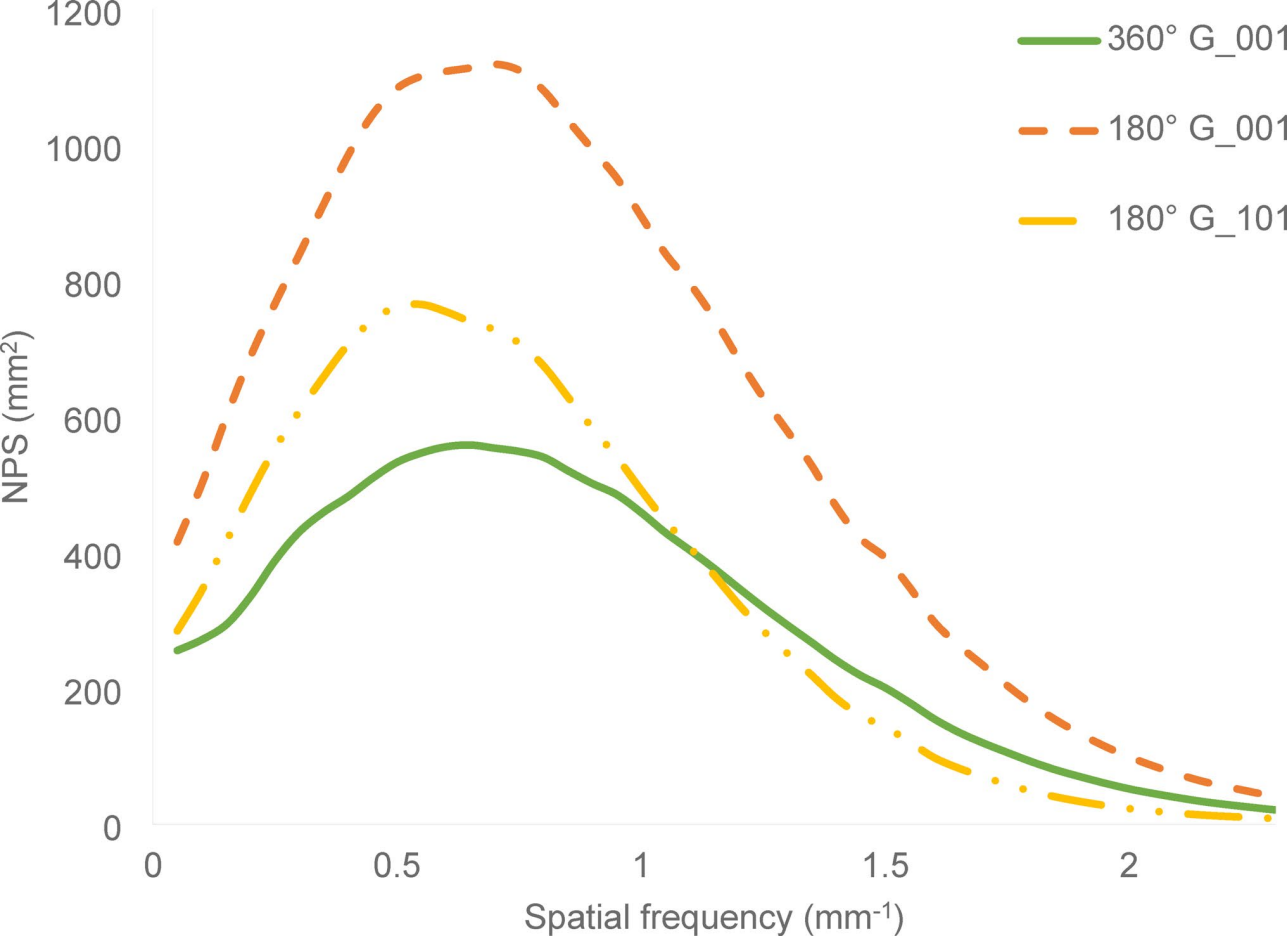
Comparison of NPS curves for 360° G_001, 180° G_001, and 180° G_101. G_001 standard reconstruction filter, G_101 low-noise filter

The NPS curves for the three scan types were derived (Fig. 3). The peak NPS values for each condition are shown in Table 2. Although the peak frequencies for the 360°(G_001) and 180°(G_001) conditions were comparable, 180°(G_101) exhibited slightly lower values. The NPS values across all frequency ranges were significantly higher for 180°(G_001) compared to 360°(G_001), whereas 180°(G_101) demonstrated markedly reduced NPS values throughout the entire frequency spectrum relative to 180°(G_001).

### Comprehensive objective image quality calculated from MTF and NPS values

**Fig. 4:**
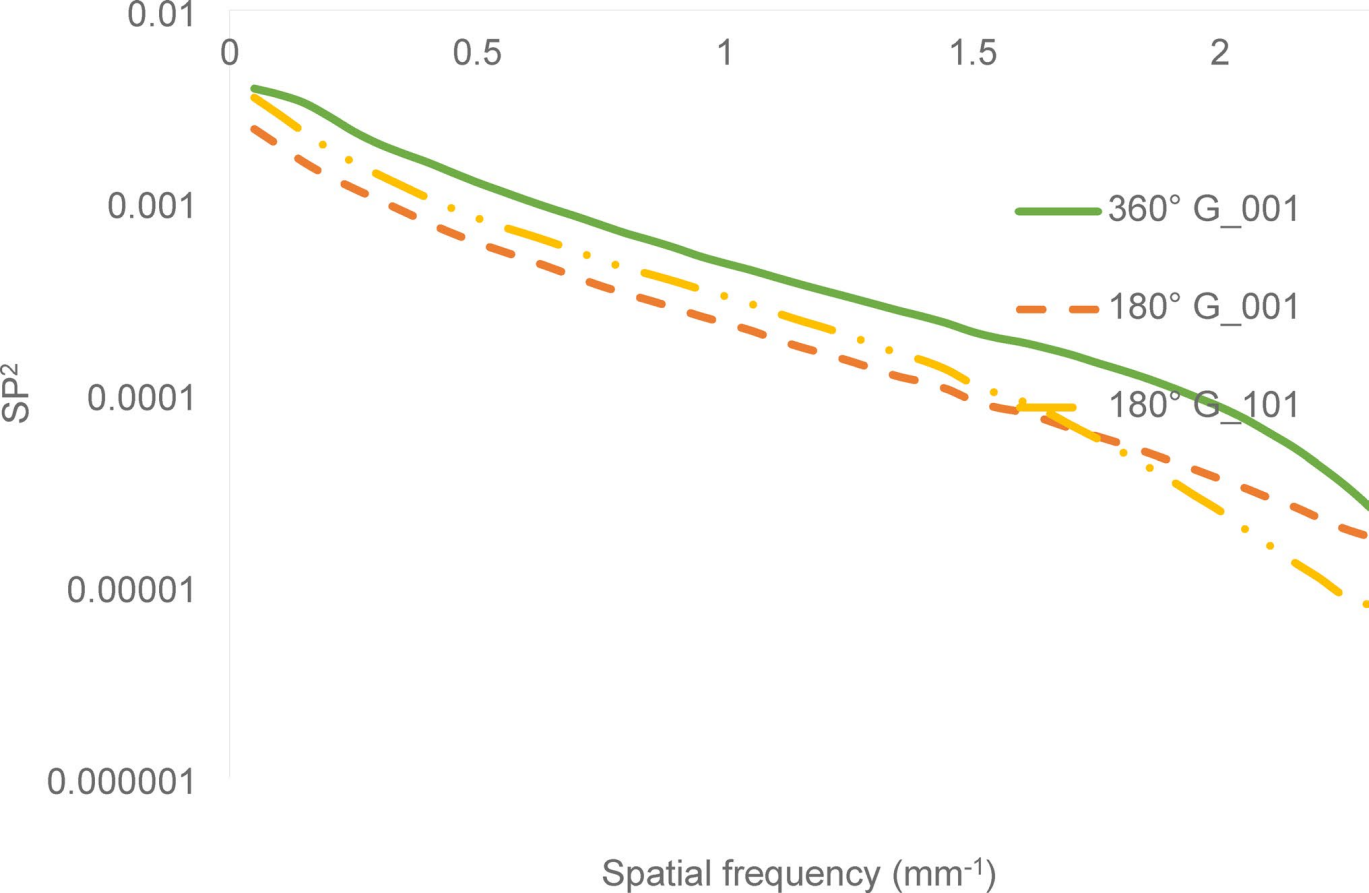
Comparison of NPS curves for 360° G_001, 180° G_001, and 180° G_101. G_001 standard reconstruction filter, G_101 low-noise filter

The SP functions for the three image sets were calculated and are shown in Fig. 4. Across all spatial frequencies, the 360°(G_001) images consistently exhibited higher SP values than both the 180°(G_001) and 180°(G_101) images. At approximately 1.73 mm^−1^, a crossover was observed, with 180°(G_101) exhibiting higher SP values than 180°(G_001) below this point, while 180°(G_001) showed higher values.

### Visual evaluation using clinical images for 360°(G_001), 180°(G_001), and 180°(G_101)

**Fig. 5:**
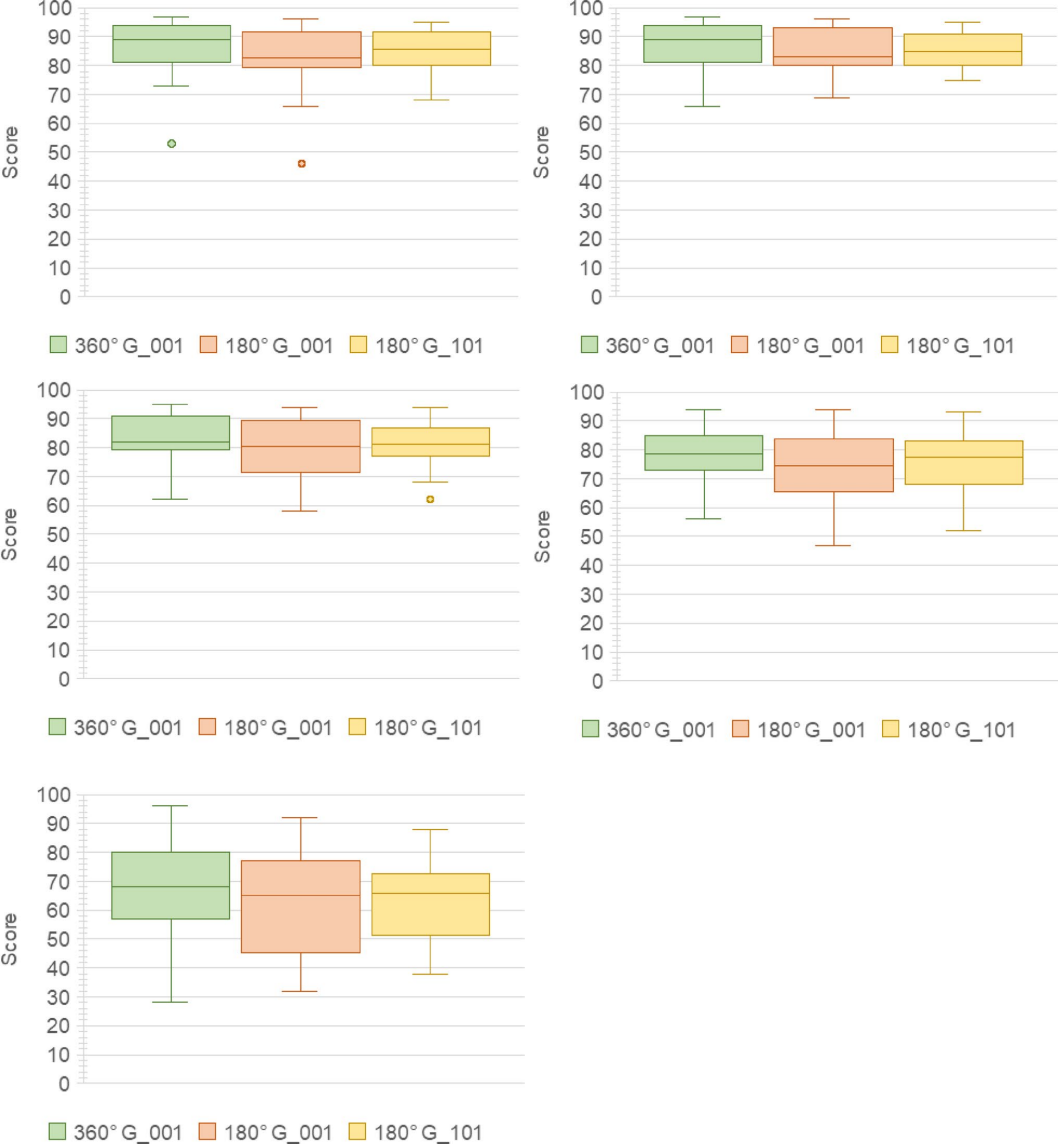
Visual evaluation (360°(G_001) vs. 180°(G_101) vs. 180°(G_101)). Evaluation items: **a** Tooth position relative to the surrounding teeth. **b** Tooth crown, **c** Tooth root. **d** Root apex of the tooth. **e** Periodontal ligament space of the tooth. Median values for (**a**): 89, 83, 86. Representative median values for (**b**)–(**e**): 89/83/85, 82/81/81, 79/75/78, and 68/65/66 (in the same order). G_001 standard reconstruction filter, G_101 low-noise filter

Figure 5 shows the results of the visual evaluation. The median scores were > 50 for all evaluation items at 360°(G_001), 180°(G_001), and 180°(G_101). The highest scores for all items were observed for 360°(G_001), followed by 180°(G_101), and 180°(G_001). The scores for the periodontal ligament space tended to be lower and exhibited greater variability than those for the other items. Tooth position relative to the surrounding teeth (*p* = 0.317), tooth crown (*p* = 0.356), tooth root (*p* = 0.372), tooth root apex (*p* = 0.632), and periodontal ligament space (*p* = 0.457) did not differ significantly between 360°(G_001) and 180°(G_001) (all *p* > 0.05). Similarly, tooth position relative to the surrounding teeth (*p* = 0.581), tooth crown (*p* = 0.362), tooth root (*p* = 0.425), root apex (*p* = 0.653), and periodontal ligament space (*p* = 0.632) did not differ significantly between 360°(G_001) and 180°(G_101) and between 180°(G_001) and 180°(G_101) (*p* = 0.818, *p* = 0.979, *p* = 0.955, *p* = 0.999, and *p* = 0.912, respectively). The ICC (1, 1) values for the three dentists were 0.64, 0.76, and 0.72, respectively, indicating moderate-to-good intra-rater reliability. The ICC (2, 3) value was 0.30, indicating poor inter-rater reliability.

**Fig. 6:**
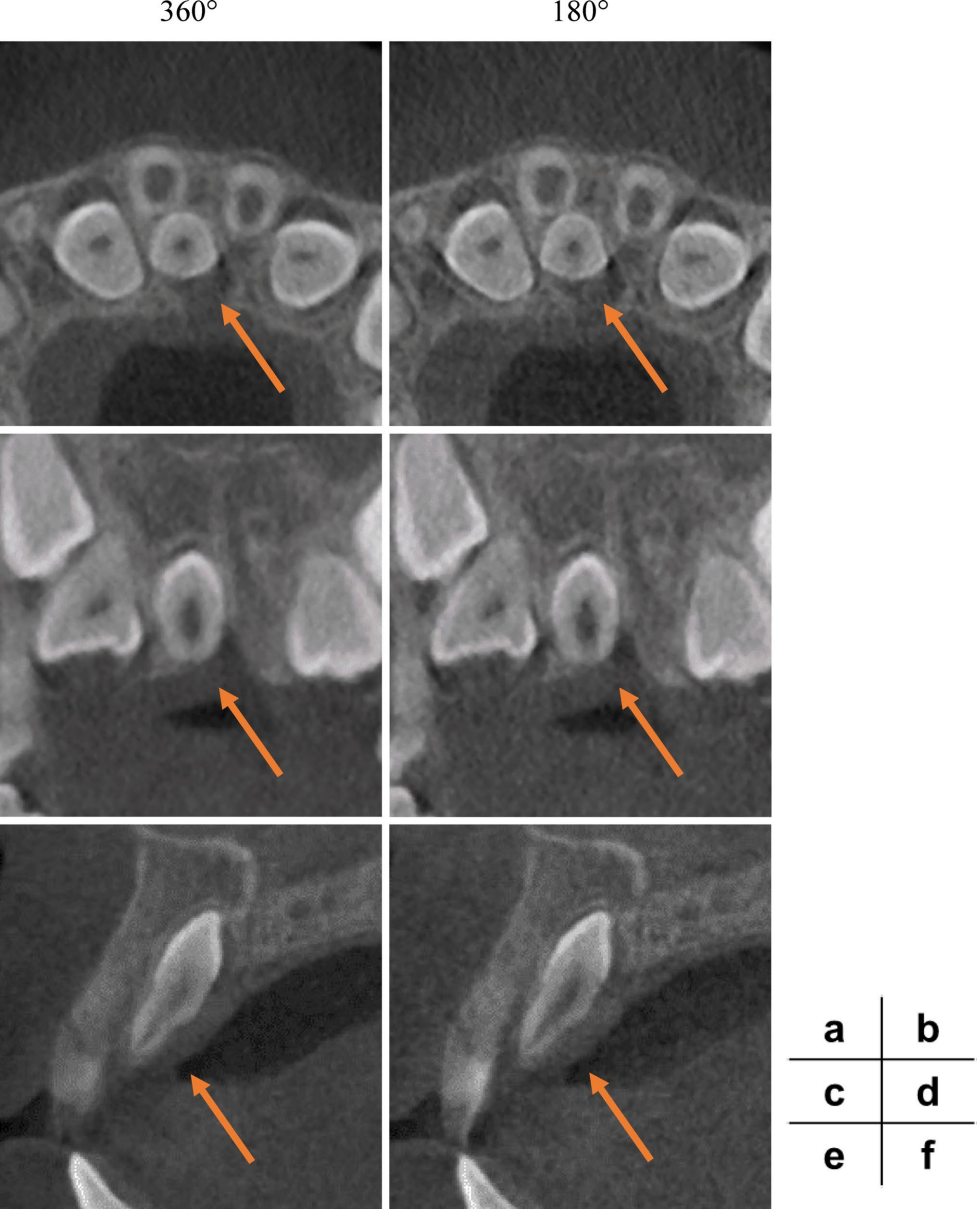
Example of images that scored higher for 180° than for 360° scan. **a** 360° scan, transverse plane; **b** 180° scan, transverse plane; **c** 360° scan, coronal plane; **d** 180° scan, coronal plane; **e** 360° scan, sagittal plane; **f** 180° scan, sagittal plane. Arrows: ectopic eruptions and impacted teeth. The 360° images show more blurring than the 180° images owing to motion artifacts. A small number of the evaluated images showed this tendency

Figure 6 presents a few cases in which the 180° image received a higher score than the 360° image. The 360° image appears slightly blurred, whereas the 180° image provides clearer structural depiction.

**Fig. 7:**
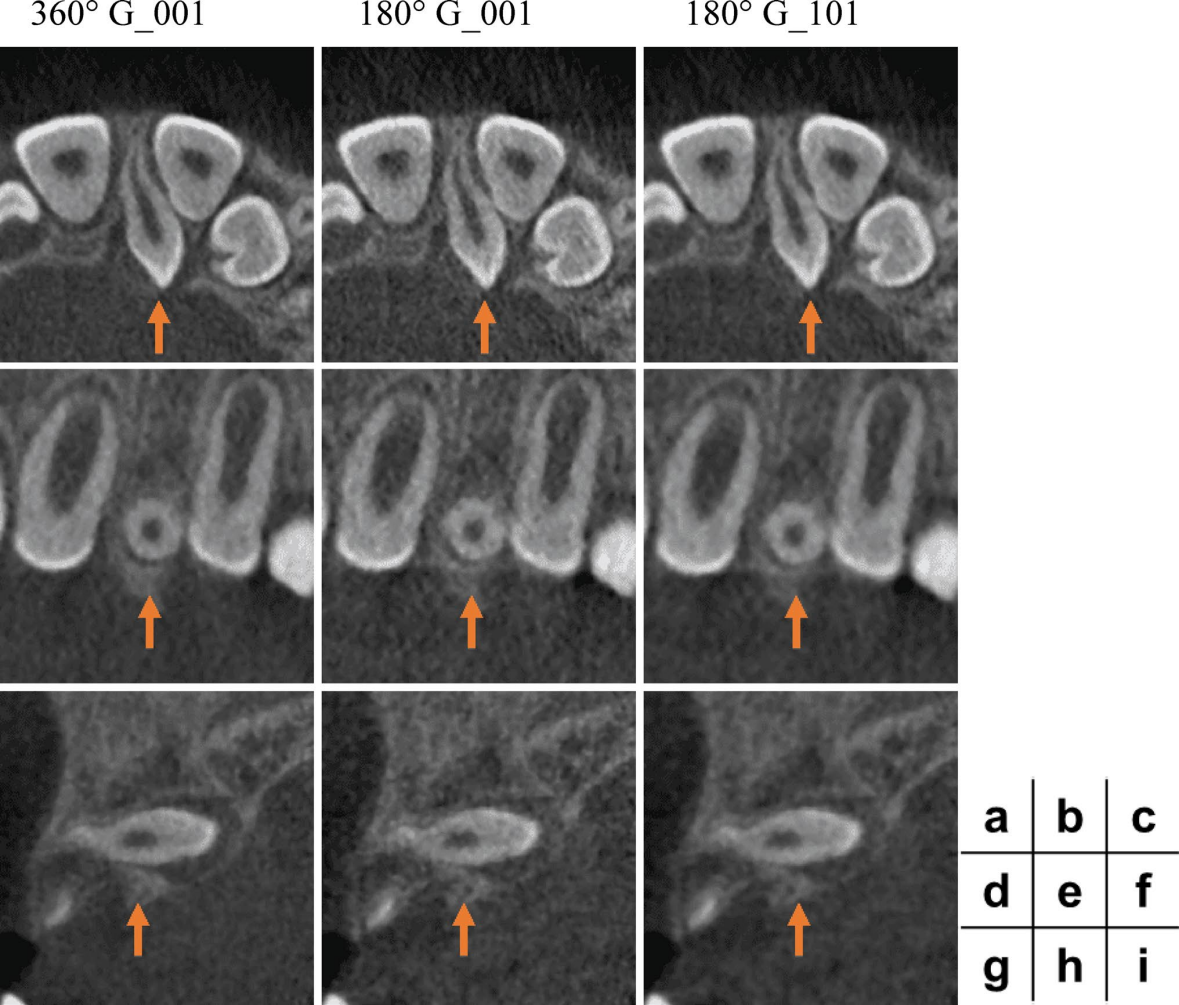
Example of clinical images used for visual evaluation (360°(G_001) vs. 180°(G_001) vs. 180°(G_101)). **a** 360°(G_001), transverse plane; **b** 180°(G_001), transverse plane; **c** 180°(G_101), transverse plane; **d** 360°(G_001), coronal plane; **e** 180°(G_001), coronal plane; **f** 180°(G_101), coronal plane; **g** 360°(G_001), sagittal plane; **h** 180°(G_001), sagittal plane; **i** 180°(G_101), sagittal plane. Arrows: ectopic eruptions and impacted teeth. Compared with the 180°(G_001), the 180°(G_101) image show reduced noise improved tooth structure, although not as much as in the 360° image. G_001 standard reconstruction filter, G_101 low-noise filter

Figure 7 shows a comparison of different reconstruction filters for 180° scan. Compared with the 180°(G_001) image, the 180°(G_101) image exhibited reduced noise and improved visibility of the tooth structures. The 360°(G_001) image provided the clearest overall visualization.

## Discussion

This study evaluated whether applying a low-noise reconstruction filter (G_101) could compensate for the increased image noise associated with 180° CBCT scanning. Although 180° scan reduces radiation exposure, it also reduces the amount of projection data, leading to increased noise that can impair the visibility of fine, low-contrast structures such as the periodontal ligament space. Our findings suggest that applying the G_101 filter improves the image quality and may provide sufficient visibility for diagnostic purposes. The results of the phantom experiments (see Supplementary Information) demonstrated that 180° images reconstructed from 360° data exhibited spatial resolution and noise characteristics comparable to those of the directly acquired 180° scans, thereby supporting the validity of the simulation approach. However, as these validations were performed under ideal conditions, further studies considering clinical factors, such as patient motion, anatomical variability, and scatter, are necessary. The periodontal ligament space is extremely narrow, measuring approximately 0.15–0.38 mm [32], corresponding to spatial frequencies of 1.3–3.3 mm^−1^. Because the periodontal ligament space contains fine structures with high-frequency components, both noise suppression and preservation of spatial resolution are essential. MTF10 values for 180°(G_101) were approximately 12% lower than those for 360°(G_001) and 10% lower than those for 180°(G_001), indicating a clear trade-off in resolution.

The results of the NPS analysis revealed higher noise across all frequencies for 180°(G_001) compared with 360°(G_001), aligning with expectations owing to limited angular coverage. Compared with 180°(G_001), 180°(G_101) effectively reduced the overall noise, particularly in the low-frequency range.

Overall, the SP function showed that 360°(G_001) provided the best overall performance. However, in the low-frequency range < 1.73 mm^−1^, 180°(G_101) outperformed 180°(G_001), indicating improved noise characteristics in that region. Although the spatial resolution was slightly reduced, the image quality of 180°(G_101) remained sufficient for diagnostic use and may be particularly beneficial in cases where reduced radiation exposure is prioritized.

The median scores in the visual evaluation were lower for the 180° images than those for the 360° images, likely because of the higher image noise inherent in limited-angle acquisition. However, all 180° images exceeded the diagnostic threshold of 50 for each evaluation item, suggesting that 180° scan is of sufficient quality to diagnose ectopic eruptions and impacted teeth in pediatric patients. This finding is consistent with those of our previous head-neck phantom study [22], and adult clinical studies such as that by Dawood et al. [33].

Interestingly, in some cases, 180° images scored higher than 360° images, likely due to reduced motion artifacts. Because 180° images were reconstructed from the posterior half of the 360° data, this implies that most patient movements occurred during anterior rotation. Although 180° scan involves fewer projections, its shorter acquisition time improves temporal resolution and may help reduce motion artifacts in pediatric patients who struggle to remain still during longer (17.5-s) 360° scans. Nevertheless, the actual reduction in motion artifacts with 180° scan was limited, suggesting that the improved temporal resolution had only a minor overall clinical impact.

In addition, low-dose and fast-scan protocols using multidetector CT (MDCT) have been proposed as alternatives to reduce motion artifacts and acquisition times. Although MDCT allows faster scanning, it typically results in higher radiation doses and lower spatial resolution than dental CBCT [34–37]. For instance, the effective dose for pediatric dental CBCT scans is approximately 30 µSv [38], whereas pediatric head MDCT examinations may involve doses of 1–2 mSv [39], an approximately 30–60-fold dose difference. Furthermore, MDCT systems are generally expensive and less accessible in dental settings. Thus, although the motion artifact reduction achieved by the proposed 180° CBCT method is limited, its primary advantage lies in maintaining a high spatial resolution while offering a relatively low-dose and cost-effective imaging option. This makes it particularly suitable for pediatric dental applications where minimizing radiation exposure and equipment costs is critical.

The median visual score for 180°(G_101) was higher than that of 180°(G_001) and lower than that of 360°(G_001); however, these differences were not statistically significant. Although this may indicate a trend toward improved visualization with the G_101 filter, the results should be interpreted with caution. Further studies with larger sample sizes or alternative evaluation methods are required to confirm the potential benefits of this reconstruction filter.

Although the image quality of the 360° scan was considered clinically acceptable, our evaluations showed no significant differences in diagnostic performance between the proposed 180° protocol, reconstructed using the G_101 filter, and the 360° scan. This suggests that the 180° protocol offers a promising low-dose alternative for pediatric dentistry, in which minimizing radiation exposure is essential. Further validation using physical phantoms and broader clinical studies is required. Although this study did not directly measure radiation doses, the scan parameters (27 mAs for 180° and 52.5 mAs for 360°) align with those in previous dosimetric studies. Ludlow et al. [38] reported effective doses of approximately 32 µSv for a 180° scan and 61 µSv for a 360° scan using the 3D Accuitomo 170 under comparable conditions. In our previous study using the same CBCT unit and imaging parameters, the measured dose area product (DAP) values were 490 mGy cm^2^ for the 360° and 249 mGy cm^2^ for the 180° protocol [22]. The corresponding air kerma values were 9.32 mGy and 4.70 mGy, respectively, indicating an approximately 50% reduction in exposure [40]. As the 3D Accuitomo F17 used in this study has similar geometry and exposure capabilities, the 180° protocol likely offers an approximately 50% dose reduction compared with the full-rotation scan. In 2011, the International Commission on Radiological Protection (ICRP) lowered the dose threshold for vision-impairing cataracts to 0.5 Gy [41–44], noting that this threshold reflects lifetime accumulated exposure, which is particularly relevant for pediatric patients who have a longer period over which dose can accumulate and whose ocular lenses are more radiosensitive [45, 46]. Limited-angle acquisition and noise-reducing reconstruction filters support both image optimization and modern radiation protection principles. On our previous phantom experiment, using the same imaging conditions as those in the present study, measured organ surface doses by attaching dosimeters to exterior of the phantom, as internal placement was not feasible [19]. The results showed that switching from 360° to 180° reduced the surface dose by 63.2% to the lens, 51.2% to the thyroid gland, 12.6% to the parotid gland, and 25.9% to the sublingual gland. These findings suggest that 180° scan may effectively lower radiation exposure across multiple radiosensitive head and neck organs, while maintaining clinically acceptable image quality.

In contrast to Houno et al. [47], who reported that noise had a minimal impact on periodontal ligament visibility using dry skull phantoms and high-frequency filters, our results demonstrated that noise suppression tend to improve visibility in pediatric clinical images. This discrepancy may be attributed to differences in voxel size, radiation dose, participant motion, and anatomical characteristics of pediatric patients. Houno et al. used phantom images derived from dry human skulls (age and sex unknown), whereas our study evaluated clinical images of pediatric patients (age 6–10 years) with ectopic eruptions and impacted teeth, where motion artifacts were present. Additionally, our protocol used a lower radiation dose and a 180° scan angle suitable for pediatric patients. We also used a smaller voxel size than that used by Houno et al. because the periodontal ligament space in impacted teeth tends to be narrower [32]. While this setting aimed to preserve fine anatomical details, it also resulted in increased image noise from the outset, further emphasizing the importance of effective noise suppression. G_101 reconstruction may improve image quality without increasing the radiation dose. Although G_101 reconstruction is specific to this device, many currently available devices also allow changes to the reconstruction filter.

One study limitation was that the visual evaluation was performed by only three dentists at a single institution. However, as these were board-certified oral and maxillofacial radiologists with considerable diagnostic experience, the results were considered reliable. In addition, the small number of patients (*n* = 12) and the low inter-observer reliability (ICC = 0.30), may limit the generalizability of the findings. Another limitation was that the findings were based on a specific CBCT system from a single vendor, which may limit the generalizability of the results to other systems. Although extensive observer training was not conducted owing to practical constraints, all observers received a brief explanation of the evaluation criteria using one randomly selected case to ensure a common understanding of the rating method. This relatively low reliability may be partly attributed to the use of a wide 0–100 scoring scale, which, although allowing for more nuanced assessments, can increase inter-rater variability.

## Conclusion

Compared with 360° scan, half-acquisition CBCT protocols showed increased noise and slight decreases in visual scores but maintained clinically acceptable image quality for the evaluation of ectopic eruptions and impacted teeth. Although 360°(G_001), 180°(G_001), and 180°(G_101), did not differ significantly, the application of a low-noise reconstruction filter (G_101) to 180° scans tended to improve image interpretability compared with the standard reconstruction. Given the substantial dose reduction and potential to mitigate motion artifacts, this combination warrants further investigation as a promising low-dose imaging strategy for pediatric dental diagnostics.

## Supplementary Information

Below is the link to the electronic supplementary material.

Supplementary Material 1
