# Supplementary material for: Pediatric dental cone-beam computed tomography using half-acquisition and low-noise reconstruction: visual evaluation of clinical images
Source: Phys Eng Sci Med. 2026 Jan 8;49(2):603–15. doi: 10.1007/s13246-025-01691-2 (PMC13375698; doi:10.1007/s13246-025-01691-2)
Supplement: Supplementary file 1 — Supplementary Material 1 [file 13246_2025_1691_MOESM1_ESM.docx]

**Supplementary Information**

**Materials and methods**

**Preliminary validation of the 180° image reconstruction method**

Before conducting the clinical visual evaluation, a preliminary phantom experiment was performed to validate the equivalence between the directly acquired 180° images and the 180° images reconstructed from the 360° data. Using the Sedentex CT-IQ phantom, CBCT scans were performed separately with 180° and 360° acquisitions. The simulated 180° images were produced by directly extracting the raw projection data for a 180° scan via the scanner console from the full 360° datasets and matching the X-ray tube rotation range of the directly acquired 180° scan, as illustrated in Fig. 1. The image quality was then objectively evaluated by comparing the modulation transfer function (MTF), which quantifies the system’s ability to maintain contrast at varying spatial frequencies and serves as a key indicator of spatial resolution in CT imaging, and the noise power spectrum (NPS), which represents the magnitude and frequency distribution of image noise, between the directly acquired 180° images and 180° images reconstructed from the 360° data.

**Spatial resolution**

The MTF was obtained using the commonly adopted wire method. A Sedentex CT-IQ cylindrical phantom (Leeds Test Objects, Boroughbridge, UK), specifically designed for dental CBCT evaluation, was used. The phantom included a cylindrical point spread function insert (35 mm diameter, 20 mm height) containing a centrally positioned stainless-steel wire 0.25 mm in diameter. The rotation center was set to the steel wire, and dental CBCT scans were performed three times for each imaging condition.

The radial virtual slit method was used to analyze the MTF. MTF values were obtained from the slice image at the center of each acquisition, as well as from slices 0.375 mm anterior and posterior to the center. In total, MTF results from nine slices were summed and averaged. The acquired image data were exported in DICOM format and analyzed using CTmeasure version 0.98f.

**Noise texture**

NPS was used to assess image noise texture. The lower section of the Sedentex CT-IQ phantom composed of uniform polymethyl methacrylate (density 1.20 ± 1.00%), was imaged. The center of rotation was set to the center of the phantom, and dental CBCT scans were performed three times for each imaging condition.

The radial frequency method was applied to analyze the NPS. A region of interest of 256×256 pixels was placed at the center of a 6×6 cm field of view (FOV) image. NPS results were obtained for 100 consecutive images (300 images in total) per scan, which were then summed and averaged. The acquired image data were exported in DICOM format and analyzed using CTmeasure version 0.98f.

**Results**

**Preliminary validation of 180° image reconstruction method**

**Spatial resolution**

Supplementary Figure 1 shows the comparison of MTF curves for directly acquired 180° images and 180° images reconstructed from 360° data**.** The 10% MTF values were approximately 1.819 mm⁻¹ for the directly acquired 180° scan images and approximately 1.875 mm⁻¹ for the 180° images reconstructed from 360° data. The MTF curves almost overlapped across the entire spatial frequency range.

**Noise texture**

Supplementary Figure 2 shows the comparison of NPS curves for directly acquired 180° images and 180° images reconstructed from 360° data**.** The peak frequencies of the NPS were approximately 0.616 mm⁻¹ for the directly acquired 180° imaging and approximately 0.649 mm⁻¹ for the 180° imaging reconstructed from 360° data. These values were very similar, and the overall NPS curves exhibited similar shapes across the spatial frequency range.

**Supplementary Figure 1** Comparison of MTF curves between directly acquired 180° images and 180° images reconstructed from 360° acquisition.

**Supplementary Figure 2** Comparison of NPS curves between directly acquired 180° Images and 180° images reconstructed from 360° acquisition.
